# Optimal dose of neostigmine antagonizing cisatracurium-induced shallow neuromuscular block in elderly patients: a randomized control study

**DOI:** 10.1186/s12871-023-02233-7

**Published:** 2023-08-10

**Authors:** Mengya Cao, Huifan Huang, Jianbin Tong, Yangwen Ou, Yan Liao

**Affiliations:** 1 Department of Anesthesiology, Third Xiangya Hospital, Central South University, Changsha, Hunan 410013 P.R. China; 2 Department of Anesthesiology, The First Affiliated Hospital of Xiamen University, Xiamen University, Xiamen, P.R. China; 3 Hunan Province Key Laboratory of Brain Homeostasis, Third Xiangya Hospital, Central South University, Changsha, Hunan 410013 P.R. China

**Keywords:** Elderly patients, Neostigmine, Optimal dosage study

## Abstract

**Background:**

Residual neuromuscular block after using neuromuscular blocking agents is a common and potentially harmful complication of general anesthesia. Neostigmine is a widely used antagonist, but its optimal dose for elderly patients is unclear.

**Objectives:**

To compare the optimal dosage and safety of neostigmine for reversing shallow residual block in elderly patients after cisatracurium-induced neuromuscular block.

**Methods:**

A randomized controlled trial was conducted in 196 elderly patients undergoing non-cardiac surgery under general anesthesia with cisatracurium. Patients were assigned to receive either no neostigmine (control group) or neostigmine at 20 µg/kg, 40 µg/kg or 50 µg/kg when train-of-four (TOF) ratio reached 0.2 at the end of surgery. The primary outcome was the time to reach TOF ratio of 0.9 after administration. Secondary outcomes included TOF ratio at 10 min after administration, postoperative nausea and vomiting, postoperative cognitive impairment and post-anesthesia care unit (PACU) stay time.

**Results:**

The time to reach TOF ratio of 0.9 in the 20 µg/kg, 40 µg/kg and 50 µg/kg groups was significantly shorter than the control group (H = 104.257, *P* < 0.01), and the time of 40 µg/kg group and 50 µg/kg group was significantly shorter than the 20 µg/kg group (*P* < 0.001). There was no significant difference between 40 µg/kg and 50 µg/kg groups (*P* = 0.249). The TOF ratio at 10 min after administration showed similar results. There were no significant differences among groups in postoperative nausea and vomiting, postoperative cognitive impairment or post-operation hospital stay.

**Conclusions:**

Timely use of neostigmine after general anesthesia in elderly patients can significantly shorten time of TOF value reaching 0.9, among which 40 µg/kg dosage may be a more optimized choice.

**Trial registration:**

this study was registered on chictr.org.cn (ChiCTR2100054685, 24/12/2021).

## Background

Neuromuscular blocking agents (NMBAs) are widely used to provide muscle relaxation for endotracheal intubation, certain modes of mechanical ventilation and surgical procedures. However, with the widespread application of neuromuscular blocking agents in general anesthesia, the residual effects have become one of the important factors of postoperative complications [1–3]. At present, the most widely used muscle relaxation antagonists include acetylcholinesterase inhibitors and sugammadex. These intermediate-acting NMBAs, such as cisatracurium, can be metabolized quickly and consequently reduce the incidence of postoperative residual neuromuscular block [4]. However, the pooled rate of residual blockade, defined as a TOF ratio less than 0.90, was 41% with Confidence Interval (25-58%) when studies using intermediate-acting NMBDs were analyzed [5], leading to significant respiratory events (e.g. severe hypoxemia, airway obstruction), pharyngeal function impairment, and even increased mortality [4, 6–9].

Previous studies have shown that the optimal dose of neostigmine for reversal of minimal NMB (TOFr = 0.5) in adults is 40 µg/kg [10]. Nevertheless, compared to the adult, the elderly changes physiologically, including a reduced glomerular filtration rate, increased body fat, decrease in lean muscle mass, and decrease in total body water [11, 12], which may alter the pharmacokinetics and metabolism of drugs. A larger dose may be required to antagonize the residual block of muscle relaxants in the elderly [13]. However, a larger dose of neostigmine may increase the occurrence of side effects such as bradycardia and increased secretions. Thus, it is urgent to recommend the optimal dose of neostigmine for antagonizing the neuromuscular blocking effect of cisatracurium in the elderly with general anesthesia. Accordingly, we aimed to explore the optimal dosage and safety of neostigmine to reverse shallow residual block from a TOF ratio of 0.2 in elderly patients.

## Methods

### Study design and patient selection

This study was approved by the Ethics Committee of the Third Xiangya Hospital. Written informed consent was obtained from all patients. Inclusion criteria were: aged 60 to 85 years, American Society of Anesthesiology (ASA) physical status 1 to 3, and scheduled for elective surgery under general anesthesia with cisatracurium for tracheal intubation. Exclusion criteria were: BMI < 18.5 kg/m^2^ or BMI ≥ 28 kg/m^2^, significant hepatic or renal dysfunction (glutamic-pyruvic transaminase/glutamic oxaloacetic transaminase > 80 U/L, creatinine > 104 umol/L), family history of malignant hyperthermia, known allergy to one of the drugs used in this protocol, or recent use of sedatives, anti-depressants.

Patients were randomly assigned into four groups according to the dose of neostigmine used, using a computer generated list of random numbers. Neuromuscular block was induced with cisatracurium and neostigmine 0 µg/kg (Normal Saline, NS), 20 µg/kg, 40 µg/kg or 50 µg/kg was administered at a TOF ratio of 0.2 to reverse the block. Anaesthesia nurses who were not involved in the care of the patients helped prepare the study drug according to randomisation.

### Procedure

On arrival at the operating room, an intravenous cannula was inserted in the forearm vein of the patient, and standard anesthesia monitoring (noninvasive blood pressure, electrocardiogram, and oxygen saturation) were established and anesthesia depth was monitored using Bispectral Index (BIS, Medtronic, Minneapolis, MN, USA). Anesthesia was induced with sufentanil (0.5 µg/kg) and etomidate (0.2–0.3 mg/kg), cisatracurium (0.15–0.2 mg/kg). Anaesthesia was maintained by a continuous inhalation of 1% sevoflurane and infusion of propofol (3–4 mg/kg/h) and remifentanil (0.5-1.0 µg/kg/min); the infusion speed was adjusted to maintain the BIS between 40 and 60. Cisatracurium was added as required to facilitate the completion of the surgery. After tracheal intubation, ventilation was controlled to maintain arterial oxygen saturation at 96% or higher and normocapnia. Body temperature was maintained at 36.0 °C or higher. Sevoflurane was stopped about 1 h before the end of the surgery and the flow of fresh gas was increased to ensure no residual of inhalational anesthetics at the end of the surgery. Ondansetron 4 mg was administered intravenously 0.5 h before the end of the surgery to prevent nausea and vomiting. An additional 10–15 µg of sufentanil was administered at the end of surgery for postoperative pain management.

Neuromuscular function was evaluated by TOF-Watch SX acceleration muscle relaxation monitor (Organon, Dublin, Ireland). After the skin had been cleaned carefully, two surface skin electrodes were attached over the ulnar nerve proximal to the wrist at a distance of 3–6 cm. After immobilising the forearm, the acceleration transducer was fixed firmly to the volar side of the distal phalanx of the thumb on a small elastic hand adapter (TOF-Watch Handadapter; Schering-Plough, Swords, Ireland). The monitoring was initiated after induction, but before cisatracurium administration. The device was calibrated automatically after a 50-Hz tetanic stimulation for 5 s and a stable baseline (< 5% change in TOF ratio) was recorded. Normalized TOF values were calculated and recorded according to baseline TOF values. TOF stimulus was applied every 60 s until a TOF ratio greater than or equal to 0.9. When a TOF ratio of 0.2 was reached two times consecutively, a pre-determined dose of neostigmine provided by Sine Jinzhu Pharmaceutical company (Shanghai, China) was administered according to the patient group (NS, 20 µg/kg, 40 µg/kg or 50 µg/kg), accompanied by glycopyrrolate in an 1:5 ratio. The time from the administration of neostigmine to a TOF ratio of 0.9 was recorded. The proportion of patients with TOF value ≥ 0.9 10 min after neostigmine administration; the incidence of nausea and vomiting in patients on postoperative day 1-day 3; the proportion of patients with Mini-Mental State Examination (MMSE) score decrease ≥ 3 points on postoperative day 3; and PACU stay time after neostigmine administration were followed up.

### Statistical analysis

A sample size was calculated based on the primary outcome variable (the time from the administration of neostigmine to a TOF ratio of 0.9). To achieve a power of 0.8 with an alpha level of 0.05 and an effect size of f = 0.25, and considering a 10% drop out rate, a total sample size of N = 200 was needed. SPSS 22.0 software was used for statistical analysis. Count data were expressed as composition ratio; normally distributed measures were expressed as mean ± standard deviation, and t-test was used for comparison between two groups and one-way ANOVA for comparison between multiple groups; non-normally distributed measures were expressed as median (interquartile range), and Kruskal-Wallis H rank sum test was used for comparison between multiple groups; while categorical data were expressed as numbers with percentages and were compared using the Chi-square test. *P* < 0.05 was considered to indicate statistical difference.

## Results

**Fig. 1:**
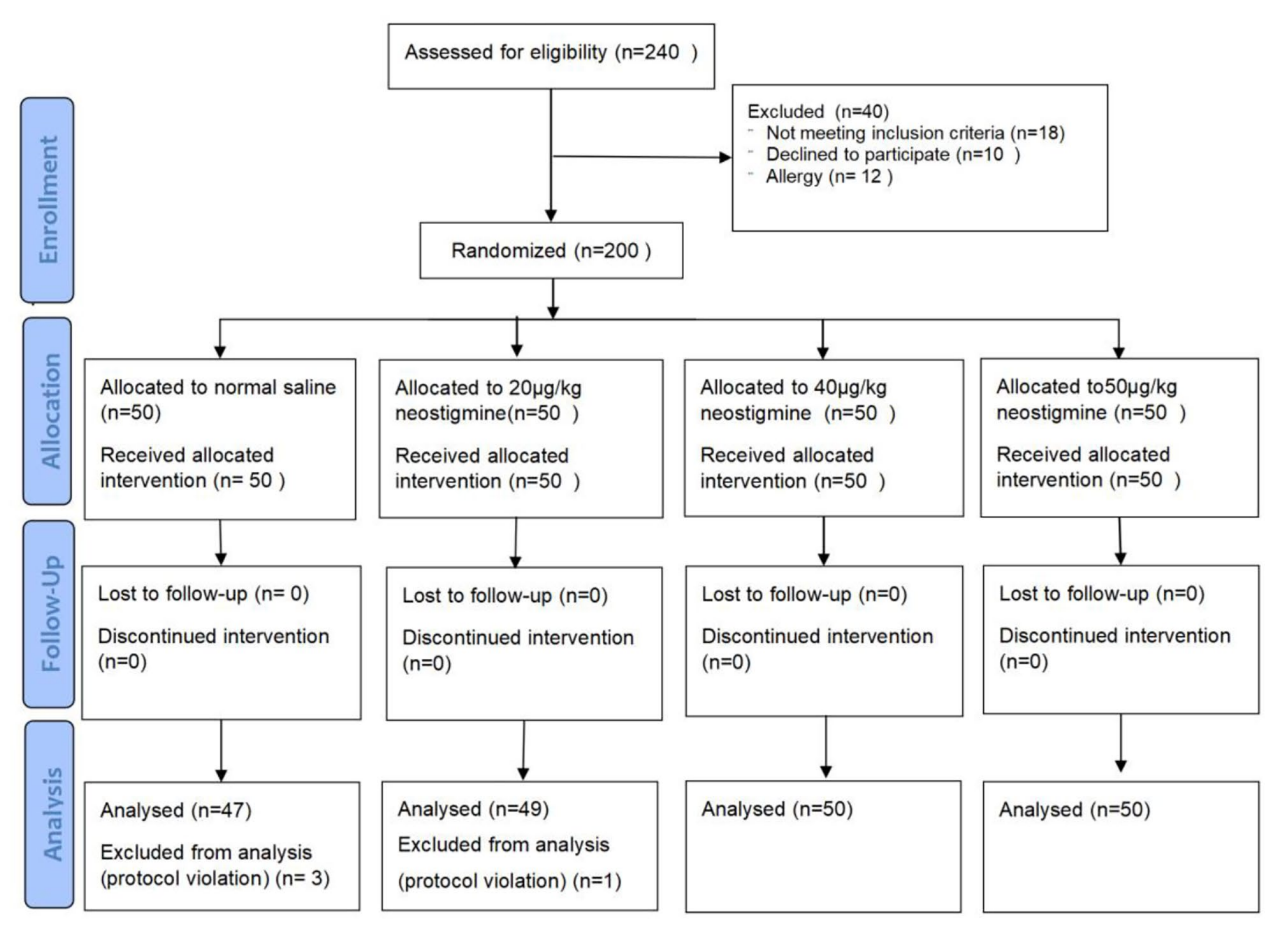
CONSORT 2010 flow diagram. (adapted from http://www.consort-statement.org/)

From January 2022 to November 2022, 240 ASA I-III patients aged from 60 to 85 who were scheduled for elective non-cardiac surgery under general anesthesia were enrolled, of which 40 patients were excluded due to contraindications (n = 18), allergies (n = 12) or declined to participate (n = 10). 200 patients completed randomization and participated in the study. 4 patients were excluded due to protocol violation (3 in NS group and 1 in 20 µg/kg group B). The number of patients included in the final statistical analysis was 196 (Fig. 1).

### Comparison of general data and perioperative data between the groups

Table 1 summarized baseline characteristics and perioperative data of patients. There were no significant differences in the age, gender, BMI between four groups (*P* > 0.05, Table 1), which means baseline characteristics were generally consistent between groups. As for the perioperative data, there was no significant difference in the amount of muscle relaxant cisatracurium, surgery duration, anesthesia duration among the four groups (*P* > 0.05, Table 1). Intergroup comparison suggests that patients from 40 µg/kg group had more blood loss than those from control group (*P* < 0.05). The type of surgery is comparable among the groups.

**Table 1:** Analysis of baseline characteristics

	Control group	Neostigmine groups				
	NS (n = 47)	20 µg/kg (n = 49)	40 µg/kg (n = 50)	50 µg/kg (n = 50)	H or X^2^	*P*
Age (y)	67 (64,70)	68 (65,71)	68 (63,71)	68 (66,73)	2.900	0.402
Gender					3.450(X^2^)	0.327
Male (%)	20 (42.6%)	23 (46.9%)	22 (44.0%)	15 (30.0%)		
Female (%)	27 (57.4%)	26 (53.1%)	28 (56.0%)	35 (70.0%)		
BMI (kg/m^2^)	23.1 (21.1,24.3)	23.15 (21.3, 25.4)	22.5 (20.0, 24.6)	22.4 (19.2, 24.8)	2.900	0.409
duration of surgery (min)	185 (140,258)	187.5 (148.75,306.5)	200 (156,265)	190 (133,247)	2.410	0.492
duration of anesthesia (min)	200 (155,300)	213.5 (174.5,322.5)	225 (175,290)	205 (153,280)	2.535	0.469
Cisatracurium (mg)	23 (18,30)	20 (14,30)	24 (16.5,38)	24 (16,30)	3.040	0.385
blood loss (ml)	100 (50,200)	100 (50,337.5)	200 (50,425)	200 (50,500)	9.776	0.021
fluid infusion (ml)	2100 (1600, 3400)	2600 (2100,3137.5)	2600 (2050,3150)	2550 (1600,3250)	5.579	0.134
T＜36.0℃ (n)	0	3	3	4		
Type of surgery (%)					12.199(X^2^)	0.379
Gynecology	9 (19.1%)	9 (18.4%)	9 (18.0%)	10 (20.0%)		
orthopedics	6 (12.8%)	11 (22.4%)	15 (30.0%)	17 (34.0%)		
Urology	7 (14.9%)	7 (14.3%)	6 (12.0%)	5 (10.0%)		
General Surgery	22 (46.8%)	22 (44.9%)	20 (40.0%)	18 (36.0%)		
Thoracic Surgery	3 (6.4%)	0	0	0		

BMI: Body Mass Index

### Comparison of results of postoperative muscle relaxation monitoring

**Fig. 2:**
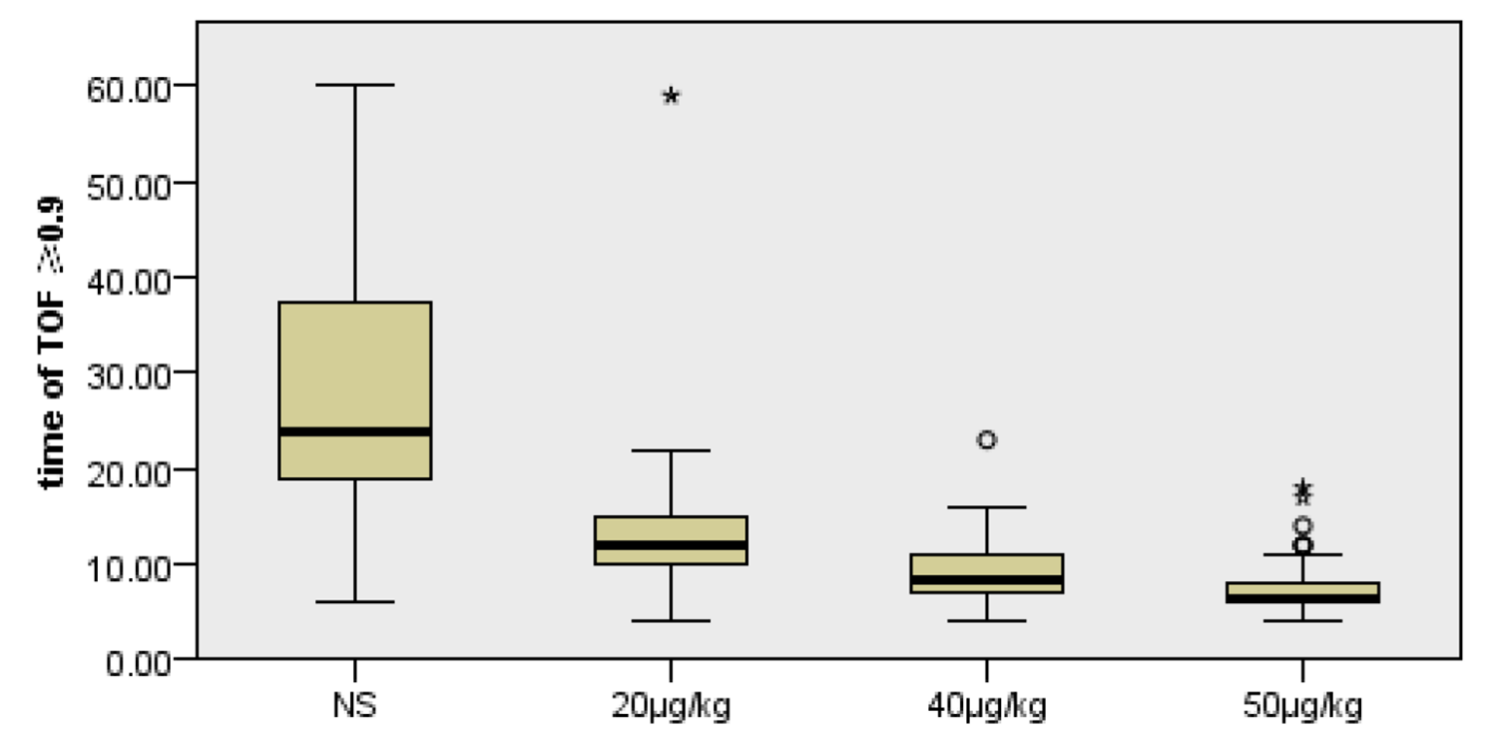
Analysis of time of TOF ≥ 90% after neostigmine (min). Time of 20 µg, 40 and 50 µg groups was significantly shorter than that in NS group after administration of neostigmine (all *P* < 0.001); Time of 40 and 50 µg groups was further significantly reduced compared with that in 20 µg (*P* = 0.032 and *P* < 0.001,respectively);yet the time between 40 and 50 µg was comparable (*P* = 0.249); *P < 0.05

As listed in Table 2, the time to reach TOF value of 0.9 after administration of neostigmine, TOF value at 10 min after administration of neostigmine and PACU stay time were significantly different among the four groups of patients (H = 104.257, *P* < 0.01). In detail, intergroup comparison showed that the time to reach TOF value of 0.9 in 20 µg, 40 and 50 µg groups was significantly shorter than that in NS group after administration of neostigmine (all *P* < 0.01); meanwhile, the time to reach TOF value of 0.9 in 40 and 50 µg groups were further significantly reduced compared with that in 20 µg group (*P* = 0.032 and *P* < 0.001, respectively), but there was no significant difference between 40 and 50 µg groups (*P* = 0.249) (Fig. 2).

**Fig. 3:**
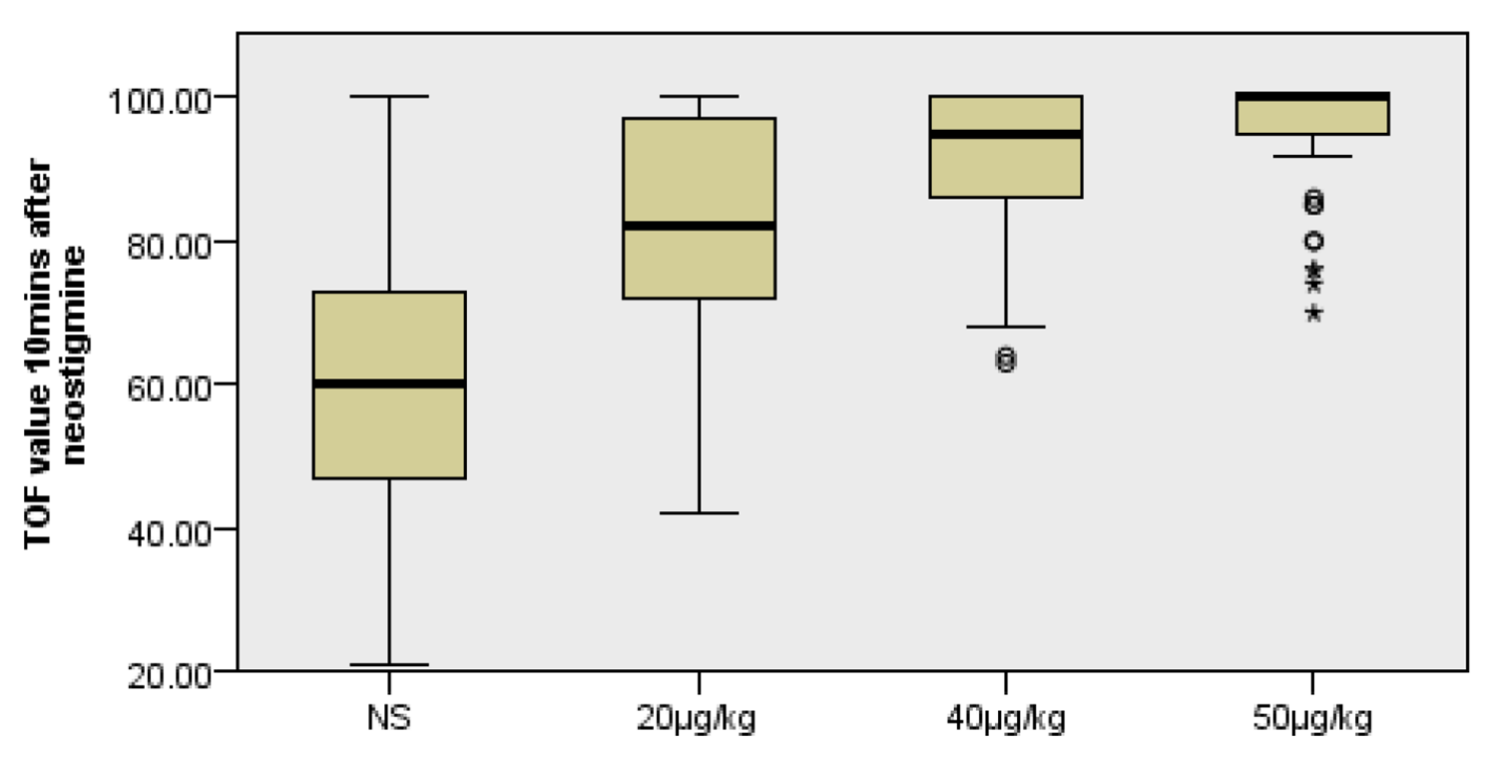
Analysis of TOF value 10 min after neostigmine (%). TOF value of 20 µg, 40 and 50 µg groups was significantly higher than that in NS group after administration of neostigmine (*P* < 0.001); value of 40 and 50 µg groups was further significantly increased compared with that in 20 µg (*P* = 0.049 and *P* < 0.001,respectively);yet the difference between 40 and 50 µg was not significant (*P* = 0.345);*P < 0.05

Similar to the results of time to reach TOF value of 0.9, TOF value at 10 min after administration in 20 µg, 40 and 50 µg groups was significantly increased compared with that in control group (H = 93.351, *P* < 0.01); meanwhile, TOF value at 10 min after administration in 40 and 50 µg groups was further significantly increased compared with that in 20 µg group (*P* = 0.049 and P < 0.001,respectively), but there was no significant difference between 40 and 50 µg groups (*P* = 0.345) (Fig. 3).

In addition, we evaluated the postoperative outcomes of each group. As shown in the Table 2, PACU stay time after neostigmine was different among the four groups (H = 10.671, *P* < 0.01). Intergroup analysis suggests that 50 µg/kg may shortern PACU stay time compared with normal saline (*P* = 0.024). We found no difference among the groups in the incidence of cognitive decline, postoperative nausea and vomiting; the length of postoperative hospital stay showed no difference either.

**Table 2:** Analysis of postoperative muscle relaxation monitoring and patient recovery

	Control group		Neostigmine groups			
	NS (n = 47)	20 µg/kg (n = 49)	40 µg/kg (n = 50)	50 µg/kg (n = 50)	H or X^2^	*P*
time of TOF ≥ 90% after neostigmine (min)	22 (18,38)	13 (10,15.8)	8 (7,11)	7 (6,8)	104.257	< 0.001*
TOF value 10 min after neostigmine (%)	60 (50,71)	80 (72,96)	96 (86.5,100)	100 (95,100)	93.351	< 0.001*
PACU stay time after neostigmine (min)	62 (45,69)	56.5 (47,65)	51 (45,69.5)	45 (35,57)	10.671	0.014*
postoperation hospital stay (d)	7 (6,10)	7 (5,9)	7 (5,11.5)	7 (6,10)	0.552	0.907
MMSE score	-1 (-2,0)	-1 (-1.75,1)	0 (-2,1)	-1 (-1,1)	0.397	0.941
PONV (n,%)	12 (25.5%)	8 (16.3%)	7 (14.0%)	13 (26.0%)	3.489 (X^2^)	0.322

*P < 0.05

MMSE: Mini-Mental State Examination; PONV: Postoperative nausea and vomiting

## Discussion

This study explored the optimal dose of neostigmine in elderly patients to reverse the TOF value to 0.9. The results indicated that the dose of 40 µg/kg may be an optimized choice and did not increase the incidence of postoperative cognitive impairment or PONV.

Incomplete neuromuscular recovery after general anesthesia is a common complication in patients after general anesthesia, with an incidence ranging from 31 to 64% [5, 14–16]. Neostigmine, as an acetylcholinesterase inhibitor, can bind to acetylcholinesterase like acetylcholine, but the binding is firm and hydrolysis is slow, which makes acetylcholinesterase lose its activity. The released neurotransmitter acetylcholine accumulates in the synaptic gap, thereby exciting skeletal muscle and achieving the effect of antagonizing non-depolarizing muscle relaxants.

Elderly patients are prone to residual muscle relaxation due to organ function degeneration and physiological changes that lead to slow metabolism of neuromuscular blockers [13]. Related guidelines and consensus also recommend intravenous administration of neostigmine for antagonism under the premise of no contraindications; however, the recommended dose is no different from that of normal adults, while large doses may lead to an increase in adverse effects of neostigmine (e.g. cause nausea, vomiting, convulsions, coma, slurred speech, anxiety, bradycardia and other symptoms). Therefore, clinical anesthesiologists maybe reduce or even avoid using neostigmine from a safety reason.

The results of this study show that neostigmine was used for antagonism at the end of surgery, and even a small dose (20 µg/kg) can significantly accelerate muscle function recovery; but 40 µg/kg can achieve the purpose of rapid reversal of muscle blockade effect, further increasing the dose (50 µg/kg) cannot significantly shorten the time for muscle recovery. It suggested that a dose of 40 µg/kg of neostigmine may be an optimized choice to reverse the effects of muscle relaxants quickly, without increasing postoperative complications.

Previous studies have explored the dose of neostigmine required for reversal of neuromuscular blockade. Preault A et al.[17] concluded that the neostigmine 20 µg/kg was sufficient to successfully reverse the TOF ratio from 0.4 to 0.9 within 10 min [9]. Similar result was also observed in another study conducted by Fuchs-Buder T [18]. However, these studies did not evaluate the postoperative outcome after the use of neostigmine, the conclusions still need to be further verified. Furthermore, consistent with our study, the results of E. S. Choi et al.[10] also showed that 40 µg/kg dosage of neostigmine may be a better choice. Nevertheless, the population of their study were adults aged 20–70 years (while our study was 60–85 years), the results may not be applicable to older patients. Meanwhile, our study also shows that in balanced anesthesia, 40 µg/kg of neostigmine is still the appropriate dosage to reverse neuromuscular blockade after adequate sevoflurane clearance.

Additionally, our study found that the use of neostigmine to antagonize muscle relaxation at the end of surgery did not significantly shorten the postoperative PACU stay time of patients, which may be affected by confounding factors such as surgical type, analgesic sedative drugs and wound drainage at the end of surgery. Subsequent further trials should limit and standardize trial conditions, reduce related confounding factors and increase sample size to clarify the role of neostigmine in shortening PACU stay.

## Conclusion

Timely use of neostigmine after general anesthesia in elderly patients can significantly shorten time of TOF value reaching 0.9, among which 40 µg/kg dosage may be a more optimized choice.

## Data Availability

The datasets used and/or analyzed during the current study are available from the corresponding author on reasonable request.
